# Neutrophils to high-density lipoprotein cholesterol ratio as a new prognostic marker in patients with ST-segment elevation myocardial infarction undergoing primary percutaneous coronary intervention: a retrospective study

**DOI:** 10.1186/s12872-022-02870-9

**Published:** 2022-10-05

**Authors:** Yun Chen, Dan Jiang, Hongmei Tao, Ping Ge, Qin Duan

**Affiliations:** 1 Department of Cardiology, The First Branch, The First Affiliated Hospital of Chongqing Medical University, 191 Renmin Rd, Yuzhong, Chongqing, 400000 People’s Republic of China; 2 Department of Cardiology, The First Affiliated Hospital of Chongqing Medical University, Chongqing, 400000 People’s Republic of China

**Keywords:** Neutrophils, High-density lipoprotein cholesterol, ST-segment elevation myocardial infarction, Inflammation

## Abstract

**Background:**

Neutrophils and high-density lipoprotein cholesterol (HDL-c) play critical roles in the pathogenesis of acute myocardial infarction. We aimed to investigate the value of neutrophils count to high-density lipoprotein cholesterol ratio (NHR) in predicting occurrence of in-hospital adverse events in ST-segment elevation myocardial infarction (STEMI) patients treated with primary percutaneous coronary intervention (PPCI).

**Methods:**

We retrospectively analyzed 532 patients who had been diagnosed with acute STEMI and treated with PPCI. Demographic and clinical data, admission laboratory parameters and NHR values were recorded. Major adverse cardiac events (MACE) were defined as stent thrombosis, cardiac rupture, cardiac arrest, ventricular aneurysm, malignant arrhythmia and cardiac death. Based on the receiver operating characteristic (ROC) analysis, all patients were divided into 2 groups based on the cut-off NHR value (NHR ≤ 11.28, NHR > 11.28). Cox regression analyses and the Kaplan–Meier survival curve were used to assess the prognostic ability of NHR in in-hospital MACE.

**Results:**

MACE was observed in 72 patients (13.5%) during in-hospital follow-up. NHR was significantly higher in MACE group compared to MACE-free group (10.93 [6.26–13.97] vs. 8.13 [5.89–11.16]; *P* = 0.001). The incidence of in-hospital MACE was significantly higher in the NHR > 11.28 group than in NHR ≤ 11.28 group (24.8% vs. 9.6%; *P* < 0.001). In multivariable Cox regression analyses, ALT, Killip III-IV and increased NHR (hazard ratio, 2.211; 95% confidence interval,1.092–4.479; *P* = 0.027) were identified as independent predictive factors of occurrence of in-hospital MACE. Higher NHR group had worse cumulative survival compared with the lower group.

**Conclusions:**

NHR value on admission, which is an easily calculated and universally available maker, may be useful in in-hospital risk classification of STEMI patients undergoing PPCI.

## Introduction

Acute myocardial infarction (AMI) is a major cause of global morbidity and mortality. Despite the improvements in reperfusion strategies, the outcome of patients with AMI remains unsatisfactory. Early risk stratification and timely interventions are significant in improving the prognosis of these patients.

Atherosclerosis and atherosclerotic plaque rupture are the main causes of STEMI. Atherosclerosis is characterized by inflammation and abnormal lipid metabolism and may cause plaque rupture in patients with certain risk factors [1–4]. Also, previous study showed that baseline inflammation status was an important factor in stent restenosis in STMI patient [5]. Neutrophils play an important role in innate immunity and atherosclerosis, particularly, the inflammatory responses in the pathogenesis of atherosclerosis [6]. In contrast, high-density lipoprotein cholesterol (HDL-c) has been associated with anti-inflammatory, antioxidant and antithrombotic effects [7]. Previous studies indicated that HDL-c was strongly associated with neutrophils in the pathogenesis of atherosclerosis, suggesting that HDL-c may regulate the function of activated neutrophils [8]. Activated neutrophils also affected the composition and function of HDL-c [9]. In a recent study, neutrophils count to high-density lipoprotein ratio (NHR) was identified as a novel factor in predicting poor long-term clinical outcomes in elderly patients with AMI [10]. However, the short-term prognostic ability of NHR has not been studied in ST-segment elevation myocardial infarction (STEMI) patients. Therefore, the present study aimed to investigate the association between NHR levels on admission with in-hospital major adverse cardiac events (MACE) in STEMI patients underwent primary percutaneous coronary intervention (PPCI).

## Materials and methods

### Study population

**Fig. 1:**
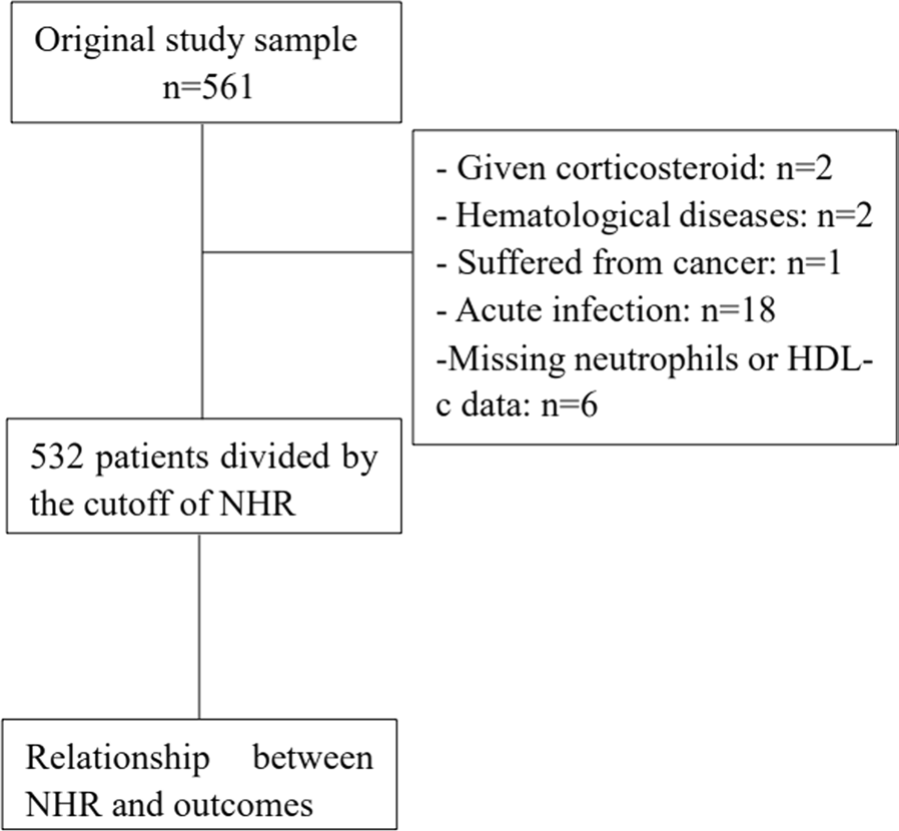
Population selection flow diagram including initial cohort and cohort exclusions. HDL-c, High-density lipoprotein cholesterol; NHR, neutrophils count to high-density lipoprotein cholesterol ratio; PCI, percutaneous coronary intervention

We retrospectively reviewed 561 STEMI patients underwent PPCI at the First Affiliated Hospital of Chongqing Medical University between September 2016 and December 2018. Participants enrolled in the study fulfilled the following criteria: (1) visited the hospital within 12 h of onset of acute myocardial ischemic symptoms and (2) ST-segment elevation of greater than or equal to 1 mm in at least 2 consecutive leads. Patients with previous myocardial infraction, a history of coronary revascularization (either coronary artery bypass graft (CABG) or PCI), active infection (including COPD and asthma), clinical evidence of cancer, chemotherapy, receiving steroid therapy for autoimmune disease, hematological proliferative disorders and patients without a laboratory data on complete blood count and cholesterol levels on admission were excluded from this study. Finally, 532 patients were included in the study (Fig. 1).

Baseline data included age, sex, systolic blood pressure (SBP) and diastolic blood pressure (DBP) on admission, smoking, hypertension, diabetes, myocardial injury markers, blood routine test, aminopherase, renal function indexes, blood lipids, high-sensitivity C-reactive protein (hs-CRP), fasting plasma glucose (FPG), HbA1c, Gensini score, NHR, Killip classification, left ventricular ejection fraction (LVEF), MACE and time of outcome events. On admission, all patients received a 12-lead electrocardiogram (ECG), routine blood test and analysis of myocardial injury markers. Other blood samples were drawn on the first day of admission. NHR was calculated as neutrophils count (×10^9^/L) to high-density lipoprotein cholesterol (mmol/L) ratio.

Hypertension was defined as use of antihypertensive drugs or SBP ≥ 140 mmHg and/or DBP ≥ 90 mmHg. While diabetes mellitus (DM) was regarded as use of antidiabetic drugs or FPG levels of greater than 7.0 mmol/L. Smoking status was assessed base on tobacco use at the time of admission. MACEs were defined as stent thrombosis, cardiac rupture, cardiac arrest, ventricular aneurysm, malignant arrhythmia and cardiac death. Cardiac rupture was diagnosed when the following criteria were fulfilled: sudden circulatory collapse and a sudden increase in pericardial effusion evident in the echocardiograms [11]. Malignant arrhythmia, including non-sustained or sustained ventricular tachycardia (VT), ventricular fibrillation (VF) and complete atrioventricular block, were recorded using ECG or cardiac monitoring. When patients experienced recurrent MACE, only the first event was considered in the analysis.

Angiotensin-converting enzyme inhibitor (ACEI) or Angiotensin receptor blocker (ARB), β-blocker and statin were administered to all patients unless there were contraindication. Patients took clopidogrel 75 mg daily or ticagrelor 90 mg twice a day and aspirin 100 mg daily after PPCI.

This study was conducted in accordance with the “Declaration of Helsinki” and approved by the institutional ethics committee (approval No. 2018-035). This study was registered on clinicaltrials.gov (approval No. NCT03527940).

### Statistical analyses

NHR was analyzed as a continuous variable and a grouped variable. The continuous variables were represented as mean ± standard deviation (SD), or median (interquartile range) based on the normality of their distribution as evaluated using the Kolmogorov–Smirnov test. The categorical variables were expressed as frequencies and percentages. The continuous variable between the two groups was compared using an independent sample *t* test or Mann–Whitney U test as appropriate. Comparisons between the categorical variables were evaluated using the Chi-Square test. Kendall’s tau-b correlation analysis was used for assessing the correlation between NHR and Killip classification, SBP. The receiver operating characteristic (ROC) curve was constructed to determine the cut-off NHR value for predicting in-hospital MACE. Univariate and multivariate Cox regression analyses were performed to identify the independent predictors of in-hospital MACE. Variables which were included into multivariable Cox regression model were statistically significant in the univariate Cox regression model. NHR was used as a grouped variable in Cox regression models. The effect of NHR on survival during hospitalization were analyzed using the Kaplan–Meier method and compared using log-rank tests. All statistical analyses were performed using SPSS Statistics software version 22.0. A two-tailed *P* value < 0.05 was considered to be statistically significant for all described analyses.

## Results

### Baseline characteristic

Of 532 participants, 72 (13.53%) patients experienced MACE, including 8 patients (1.50%) who died during hospital stay. The baseline and clinical characteristics of the patient were shown in Table 1. Patients who were MACE-free were younger than patients who suffered from MACE. Neutrophils count was higher and HDL-c was lower in the MACE group compared with the MACE-free group. NHR in the MACE group was significantly higher than that in MACE-free group (10.93 [6.26–13.97] vs. 8.13 [5.89–11.16]; *P* = 0.001). The MACE group had a worse heart function compared with the MACE-free group, as shown by a lower LVEF and a higher proportion of patients with Killip class III and IV, as well as significantly lower SBP and DBP in the MACE group.

**Table 1:** Baseline characteristics of the study population

Variable	MACE-free (n = 460)	MACE (n = 72)	*P*
Age (y)	63 (53–72)	66 (60–75)	0.014
Male/female (n)	372/88	52/20	0.124
SBP on admission (mmHg)	125 (108–145)	117 (98–136)	0.003
DBP on admission (mmHg)	75 (66–88)	70 (61–82)	0.007
Smoking (n, %)	323 (70.2)	46 (63.9)	0.279
Hypertension (n, %)	245 (53.3)	31 (43.1)	0.107
Diabetes (n, %)	148 (32.2)	22 (30.6)	0.784
MYO (ng/ml)	500 (209–500)	500 (177–500)	0.493
CK-MB (µg/L)	27.6 (4.8–65.5)	13.9 (4.2–61.2)	0.406
cTn I (ng/ml)	3.61 (0.28–16.20)	1.32 (0.22–17.1)	0.923
WBC (×10^9^/L)	10.69 (8.70–12.89)	11.90 (9.42–16.85)	0.002
RBC (×10^12^/L)	4.54 (4.15–4.90)	4.47 (4.15–4.92)	0.603
Hemoglobin (g/l)	138 (126–150)	139 (127–152)	0.978
platelet count (×10^9^/L)	189 (153–234)	189 (142–236)	0.624
Neutrophil count (×10^9^/L)	8.97 (7.00–11.02)	9.97 (7.86–13.82)	0.001
Lymphocyte count (×10^9^/L)	1.13 (0.79–1.57)	1.16 (0.81–1.70)	0.472
Monocyte count (×10^9^/L)	0.42 (0.30–0.55)	0.48 (0.34–0.70)	0.043
ALT (U/L)	40 (28–55)	58 (41–93)	< 0.001
AST (U/L)	99 (45–215)	178 (61–366)	0.004
Urea nitrogen (mmol/L)	5.7 (4.8–6.8)	5.8 (4.8–7.4)	0.357
Creatinine (µmol/L)	72 (62–85)	81 (66–100)	0.001
UA (µmol/L)	346 (285–397)	362 (295–431)	0.096
TC (mmol/L)	4.44 (3.81–5.18)	4.26 (3.52–4.98)	0.247
TG (mmol/L)	1.55 (1.00–2.27)	1.49 (0.94–2.12)	0.426
LDL-c (mmol/L)	2.92 (2.29–3.54)	2.74 (2.12–3.56)	0.224
HDL-c (mmol/L)	1.09 (0.93–1.30)	1.03 (0.88–1.29)	0.492
hs-CRP (mg/L)	4.95 (2.04–10.19)	6.67 (2.87–19.57)	0.005
FPG (mmol/L)	6.7 (5.8–8.3)	8.0 (6.9–9.9)	< 0.001
HbA1c (%)	6.0 (5.7–6.8)	6.2 (5.8–6.8)	0.128
Gensini score	72 (39–89)	80 (44–97)	0.070
Killip III-IV (%)	31 (6.7)	39 (55.7)	< 0.001
LVEF (%)	57 (52–60)	55 (45–60)	0.012
NHR	8.13 (5.89–11.16)	10.93 (6.26–13.97)	0.001

ALT, Alanine transaminase; AST, aspartate aminopherase; CK-MB, creatine kinase isoenzyme; cTn I, cardiac troponin I; DBP, diastolic blood pressure; FPG, fasting plasma glucose; HDL-c, high-density lipoprotein cholesterol; hs-CRP, high-sensitivity C-reactive protein; HbA1c, hemoglobin A1c; LDL-c, low-density lipoprotein cholesterol; LVEF: left ventricular ejection fraction; MACE, major adverse cardiac events; MYO, myoglobin; NHR neutrophils count to high-density lipoprotein cholesterol ratio; RBC, red blood count; SBP, systolic blood pressure; TC, total cholesterol; TG, triglyceride; UA, uric acid; WBC, white blood count

The age, WBC, neutrophil count, monocyte count, ALT, AST, creatinine, hs-CRP and FPG were higher in the MACE group compared with the MACE-free, but there was no significant difference in the other parameters between the two groups.

### Prognostic ability of NHR for in-hospital outcomes

**Fig. 2:**
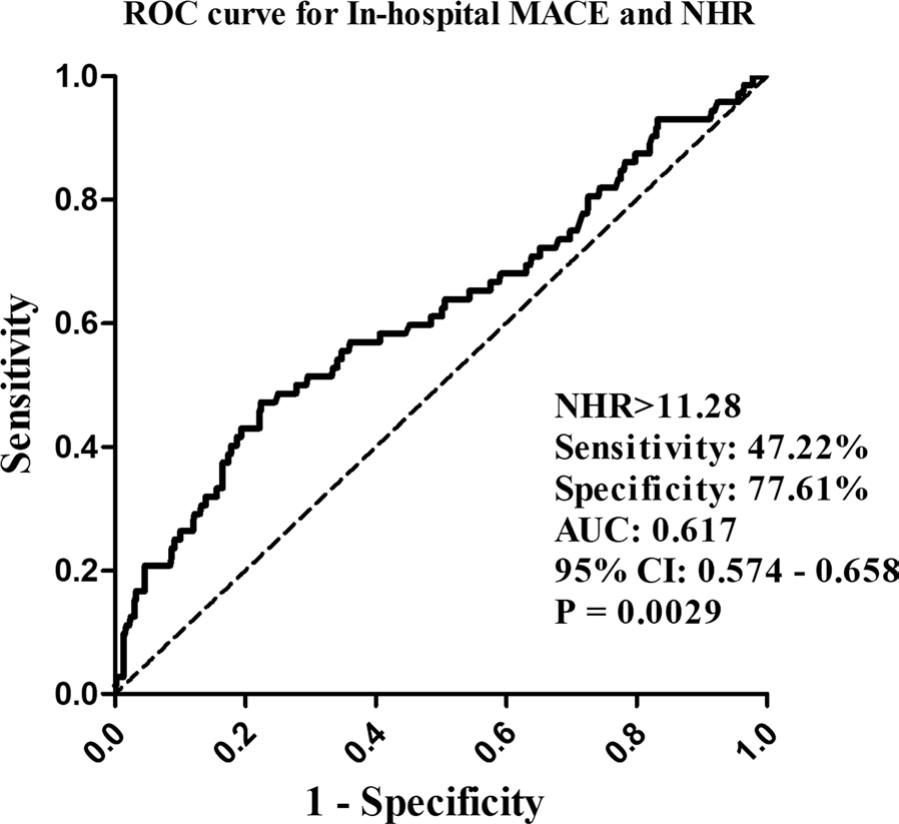
The ROC curve of NHR for predicting in-hospital MACE. The cutoff value of was NHR > 11.28 with a sensitivity of 47.22% and a specificity of 77.61%, respectively. AUC, Area under the curve; MACE, major adverse cardiac events; NHR, neutrophils count to high-density lipoprotein cholesterol ratio; ROC, receiver operating characteristic; 95% CI, 95% confidence interval

The area under the curve (AUC) of NHR for predicting in-hospital MACE was 0.617 (*P* = 0.0029, 95% confidence interval [CI]: 0.574–0.658) and the optimal cut-off point was observed at 11.28 with a sensitivity of 47.22% and a specificity of 77.61% (Fig. 2). According to the cut-off value, all patients were grouped into 2 groups based on the cut-off value (the higher group NHR > 11.28, the lower group NHR ≤ 11.28). Baseline characteristics of two groups were showed in Table 2. The incidence of MACE was higher in NHR > 11.28 group compared with NHR ≤ 11.28 group, while HDL-c levels were higher and neutrophils count lower in NHR ≤ 11.28 group. The NHR > 11.28 group had a higher proportion of patients with Killip class III and IV. Patients in NHR > 11.28 group had higher smoking proportion, MYO, CK-MB, WBC, RBC, hemoglobin, platelet count, lymphocyte count, monocyte count, aminotransferase, creatinine, UA, TG, FPG and HbA1c as well as significantly lower age, SBP, TC and LDL-c than the NHR ≤ 11.28 group. There were no significant differences in all the other parameters between the two groups.

**Table 2:** Baseline characteristics of two NHR groups of patients with STEMI

Variable	NHR ≤ 11.28 (n = 395)	NHR > 11.28 (n = 137)	*P*
Age (y)	65 (57–74)	57 (50–65)	< 0.001
Male/female (n)	298/97	126/11	< 0.001
SBP on admission (mmHg)	125 (108–144)	119 (102–141)	0.029
DBP on admission (mmHg)	74 (66–86)	72 (64–89)	0.523
Smoking (n, %)	259 (65.6%)	110 (80.3%)	0.001
Hypertension (n, %)	214 (54.2%)	62 (45.3%)	0.072
Diabetes (n, %)	125 (31.6%)	45 (32.8%)	0.795
MYO (ng/ml)	500.0 (170.0–500.0)	500.0 (359.0–500.0)	0.001
CK-MB (µg/L)	22.9(3.4–61.2)	36.8 (7.5–68.8)	0.010
cTn I (ng/ml)	2.51 (0.19–15.90)	5.55 (0.56–17.24)	0.055
WBC (×10^9^/L)	9.77 (8.37–11.60)	14.11 (12.29–17.05)	< 0.001
RBC (×10^12^/L)	4.49 (4.09–4.83)	4.64 (4.31–5.13)	< 0.001
Hemoglobin (g/l)	136 (125–147)	143 (131–159)	< 0.001
platelet count (×10^9^/L)	183 (148–228)	210 (174–255)	< 0.001
Neutrophil count (×10^9^/L)	8.12 (6.67–9.74)	12.32(10.46–14.62)	< 0.001
Lymphocyte count (×10^9^/L)	1.09 (0.75–1.54)	1.34 (0.87–1.71)	0.002
Monocyte count (×10^9^/L)	0.39 (0.29–0.53)	0.51 (0.39–0.72)	< 0.001
ALT (U/L)	38 (28–55)	50 (38–73)	< 0.001
AST (U/L)	90 (44–210)	156 (68–292)	< 0.001
Urea nitrogen (mmol/L)	5.8 (4.8–7.0)	5.6 (4.8–6.7)	0.188
Creatinine (µmol/L)	71 (61–85)	76 (66–93)	0.007
UA (µmol/L)	342 (283–402)	372 (302–438)	< 0.001
TC (mmol/L)	4.48 (3.81–5.22)	4.22 (3.50–4.79)	0.001
TG (mmol/L)	1.42 (0.94–2.12)	1.86 (1.30–2.88)	< 0.001
LDL-c (mmol/L)	2.90 (2.29–3.59)	2.77 (2.13–3.24)	0.013
HDL-c (mmol/L)	1.18 (1.02–1.38)	0.86 (0.74–0.99)	< 0.001
hs-CRP (mg/L)	5.19 (2.00–11.29)	5.20 (2.75–11.99)	0.334
FPG (mmol/L)	6.7 (5.8–8.3)	7.3 (6.1–8.7)	0.006
HbA1c (%)	6.0 (5.7–6.7)	6.2 (5.8–7.0)	0.024
Gensini score	73 (39–90)	76 (43–90)	0.527
Killip III-IV (%)	43 (10.9%)	27 (19.9%)	0.008
LVEF (%)	56 (52–60)	57 (52–60)	0.699
MACE (n, %)	38 (9.6%)	34 (24.8%)	< 0.001

Abbreviations as in Table 1

NHR was significantly associated with Killip class III-IV (r = 0.089, *P* = 0.012) and SBP (r = -0.078, *P* = 0.029).

### Univariate and multivariate Cox regression analyses

In the univariate Cox regression analyses, age, BP on admission, WBC, neutrophils count, aminotransferase, UA, hs-CRP, FPG, LVEF, Killip III-IV and NHR were associated with a high risk of in-hospital MACE. However, in multivariate regression analyses, only ALT (HR: 1.017, 95% CI: 1.008–1.026, *P* < 0.001), Killip III-IV (HR: 5.745, 95% CI: 2.795–11.807, *P* < 0.001) and NHR (HR: 2.211, 95% CI: 1.092–4.479, *P* = 0.027) were found to be independent predictors of in-hospital MACE after adjustment for other confounding variables (Table 3).

**Table 3:** Significant predictors of in-hospital MACE in univariable and multivariable Cox regression analyses

	Univariable Cox regression analysis		Multivariable Cox regression analysis	
Variable	HR (95%CI)	*P*	HR (95%CI)	*P*
Age	1.020 (1.001–1.040)	0.041	1.015 (0.988–1.043)	0.266
Male gender	0.672 (0.401–1.128)	0.133		
SBP on admission	0.982 (0.971–0.992)	< 0.001	0.995 (0.973–1.017)	0.639
DBP on admission	0.974 (0.958–0.990)	0.001	1.002 (0.967–1.038)	0.919
Smoking	0.806 (0.498–1.305)	0.380		
Hypertension	0.685 (0.429–1.093)	0.113		
Diabetes	0.886 (0.537–1.465)	0.638		
MYO	1.000 (0.998–1.001)	0.499		
CK-MB	0.997 (0.990–1.004)	0.424		
cTn I	1.000 (0.980–1.023)	0.975		
WBC	1.155 (1.092–1.221)	< 0.001	0.976(0.891–1.068)	0.594
RBC	0.803 (0.571–1.128)	0.205		
Hemoglobin	0.998 (0.987–1.008)	0.687		
platelet count	1.001 (0.997–1.004)	0.757		
Neutrophil count	1.160 (1.093–1.231)	< 0.001		
Lymphocyte count	1.192 (0.874–1.624)	0.267		
Monocyte count	1.310 (0.962–1.784)	0.086		
ALT	1.016 (1.012–1.020)	< 0.001	1.017 (1.008–1.026)	< 0.001
AST	1.002 (1.001–1.003)	< 0.001	0.998 (0.996–1.000)	0.051
Urea nitrogen	1.018 (0.959–1.080)	0.560		
Creatinine	1.002 (0.999–1.004)	0.135		
UA	1.003 (1.000–1.005)	0.027	1.002 (0.999–1.005)	0.181
TC	0.875 (0.707–1.083)	0.220		
TG	0.995 (0.887–1.115)	0.926		
LDL-c	0.837 (0.650–1.078)	0.169		
HDL-c	0.704 (0.334–1.482)	0.355		
hs-CRP	1.052 (1.018–1.087)	0.002	0.982 (0.939–1.028)	0.446
FPG	1.091 (1.034–1.151)	0.001	0.981 (0.904–1.065)	0.650
HbA1c	1.014 (0.885–1.161)	0.844		
Gensini score	1.004 (0.999–1.009)	0.103		
Killip III-IV	10.421 (6.486–16.743)	< 0.001	5.745 (2.795–11.807)	< 0.001
LVEF	0.960 (0.932–0.988)	0.006	0.988(0.950–1.028)	0.549
NHR > 11.28	2.777 (1.748–4.412)	< 0.001	2.211(1.092–4.479)	0.027

HR, Hazard ratio; other abbreviations as in Table 1 and 2

### Survival analysis

**Fig. 3:**
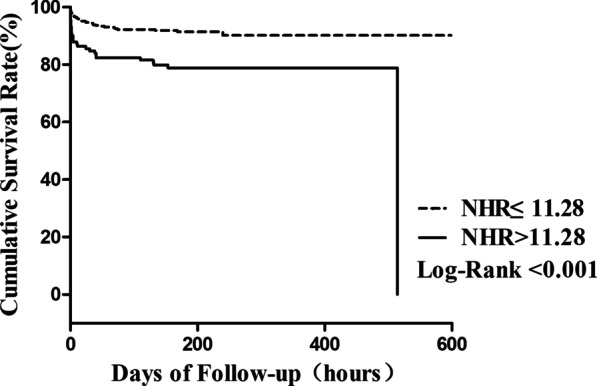
Kaplan–Meier survival curves of in-hospital MACE according to NHR (log-rank test: *P* < 0. 001). MACE, Major adverse cardiac events; NHR, neutrophils count to high-density lipoprotein ratio

The Kaplan–Meier curve were plotted base on the event free survival data from the in-hospital follow-up. As showed in Fig. 3, patients in the NHR > 11.28 group had a worse clinical outcome than patients in the NHR ≤ 11.28 group.

## Discussion

The main finding of the study were that ALT, presence of Killip class III-IV and NHR value was independently associated with in-hospital MACE in STEMI patients treated with PPCI. In addition, increased NHR was significantly associated with worse Killip classification. To the best of our knowledge, this was the first study focused on the prognostic value of NHR in predicting MACE in STEMI patients.

Inflammation plays a critical role in formation of atherosclerosis. In the presence of serval risk factors, inflammation may lead plaque rupture, which is the main cause of STEMI. Also, inflammation is correlated with adverse event and life threating complications in these patients. Various inflammatory indexes, such as blood platelet count, CRP, neutrophil to lymphocyte ratio, and so on, have been investigated in patients with cardiovascular disease and demonstrated to be short or long term prognostic factors [12–14]. In the current study, admission WBC, neutrophil count, monocyte count, hs-CRP level were significantly higher in the MACE group compared with the MACE-free.

Neutrophils, as a component of immune cell, have attracted the attention of researchers in the past decade, emerging studies have suggested that neutrophils play an important role in different stages of atherosclerosis, including atherogenesis, plaque destabilization, plaque erosion, as well as repair processes in myocardial infarction[15–17]. When arterial plaque ruptured, neutrophils and platelets aggregated rapidly at the lesion site, where the neutrophils may induce and amplify platelet activation and blood coagulation [18–20].Neutrophil extracellular trap, a protein synthesized by neutrophils, has been considered to be a key component of pathological thrombi and drive cardiovascular disease [21, 22]. Neutrophil-derived micro-vesicles represented another mechanism by which neutrophils amplify inflammatory processes [23]. In addition, Clinical studies have shown that the increase in neutrophils was independently correlated with the infarct size, left cardiac function and adverse events in patients with AMI [24, 25].

HDL-c is a well-known antioxidant, anti-inflammatory, antiapoptotic, antithrombotic, anti-atherosclerotic agent that also has reverse-cholesterol transport effects [26]. Reduced HDL-c is common in patients with AMI and is associated with the severe coronary artery disease. Clinical studies showed HDL-c was negatively correlated with the risk of coronary heart disease, and lower levels of HDL-c were an independent predictor of adverse cardiovascular events [27]. In our study, admission HDL-c levels were lower in the MACE group but did not show statistical significance and it did not correlate with in-hospital MACE in multivariate analysis.

Recent studies on HDL-c have focused on its immunomodulatory effects [28]. Neutrophils can be activated in hyperlipidemia. HDL-c has been shown to stimulate the biogenesis of microRNA-223-3p which regulates neutrophil development, hyperactivity and recruitment during infection [29]. Another study indicated that HDL may contribute to the neutrophil activation by decreasing neutrophil membrane lipid rafts [30]. In in vitro and in vivo studies of myocardial ischemia/reperfusion injury, HDL-c administration prior to ischemia or reperfusion had a multidimensional protective effect on cardiac function, and involved the ability of HDL-c to restrict endothelial permeability, as well as promote vasodilation and neovascularization in coronary endothelial cells [31, 32].

As mentioned above, NHR was considered as a valuable biomarker of inflammatory status and may be a predictor for adverse cardiovascular events in myocardial infarction patient. A recent clinical study on elderly patients with AMI by Huang et al. [10] reported that high NHR was a predictor of the long-term clinical outcomes including mortality and recurrence of myocardial infraction, and was superior to pre-existing biomarker called monocyte to High-density Lipoprotein Cholesterol ratio (MHR) and low-density Lipoprotein Cholesterol to High-density Lipoprotein Cholesterol ratio (LDL-C/HDL-C). The present study was conducted to further verify the short-term prognostic value of NHR in STEMI patients. In the study, NHR was higher in patients with in-hospital MACE than those patients who were MACE-free. ROC curve analysis revealed that NHR greater than 11.28 as a cutoff value for in-hospital MACE. A high NHR value was independently associated with increased incidence of in-hospital MACE in STEMI patients treated with PPCI. Patients with NHR greater than 11.28 groups had a 2.211 (95% CI: 1.092–4.479)-fold increased risk of occurrence of in-hospital MACE, compared with NHR less than 11.28.

Interestingly, patients with increased NHR in our study were associated with worse Killip classification and SBP, which indicated that NHR may be a new biomarker of cardiac function. This funding was consistent with a previous report which suggested that excessive infiltration or delayed regression of neutrophils aggravated myocardium injury via the abundant release of inflammatory mediators and proteinases [33].

There were some limitations in this study. First, the retrospective design of the study set a limit to the convincement of our study. The second limitation was this research was based in a single center and the study population size was small, so multicenter and large-scale studies are needed to verify this conclusion.

In conclusion, NHR is an easily calculated and universally available marker that may be useful in in-hospital risk classification of STEMI patients undergoing PPCI. Given the interactions and significant roles played by neutrophils and HDL-c in the pathogenesis of atherosclerosis and myocardial infraction, strategies aimed at reducing the NHR have potential therapeutic effects for STEMI patients undergoing PPCI.

## Data Availability

The datasets used and/or analyzed during the current study are available from the corresponding author on reasonable request.

## Abbreviations

AMI: Acute myocardial infarction
ACEI: Angiotensin-converting enzyme inhibitor
ARB: Angiotensin receptor blocker
AUC: Area under the curve
CABG: Coronary artery bypass graft
DBP: Diastolic blood pressure
DM: Diabetes mellitus
ECG: Electrocardiogram
FPG: Fasting plasma glucose
hs-CRP: High-sensitivity C-reactive protein
HDL-c: High-density lipoprotein cholesterol
LDL-C/HDL-C: Low-density lipoprotein cholesterol to high-density lipoprotein cholesterol ratio
LVEF: Left ventricular ejection fraction
MACE: Major adverse cardiac events
MHR: Monocyte to high-density lipoprotein cholesterol ratio
NHR: Neutrophils count to high-density lipoprotein cholesterol ratio
PPCI: Primary percutaneous coronary intervention
ROC: Receiver operating characteristic
SD: Standard deviation
STEMI: ST-segment elevation myocardial infarction
SBP: Systolic blood pressure
VT: Ventricular tachycardia
VF: Ventricular fibrillation
